# The PREdictor of MAlnutrition in Systemic Sclerosis (PREMASS) Score: A Combined Index to Predict 12 Months Onset of Malnutrition in Systemic Sclerosis

**DOI:** 10.3389/fmed.2021.651748

**Published:** 2021-03-17

**Authors:** Gianluca Bagnato, Erika Pigatto, Alessandra Bitto, Gabriele Pizzino, Natasha Irrera, Giuseppina Abignano, Antonino Ferrera, Davide Sciortino, Michelle Wilson, Francesco Squadrito, Maya H. Buch, Paul Emery, Elisabetta Zanatta, Sebastiano Gangemi, Antonino Saitta, Franco Cozzi, William Neal Roberts, Francesco Del Galdo

**Affiliations:** ^1^National Institute for Health Research (NIHR) Leeds Biomedical Research Centre (BRC), Leeds Teaching Hospitals National Health Service (NHS) Trust and Leeds Institute of Rheumatic and Musculoskeletal Medicine, University of Leeds, Leeds, United Kingdom; ^2^Department of Clinical and Experimental Medicine, University of Messina, Messina, Italy; ^3^Department of Medicine, Villa Salus Hospital, Venice, Italy; ^4^Rheumatology Institute of Lucania (IReL), Rheumatology Department of Lucania, San Carlo Hospital of Potenza and Madonna delle Grazie Hospital of Matera, Potenza, Italy; ^5^Centre for Musculoskeletal Research, School of Biological Sciences, Faculty of Biology, Medicine & Health, University of Manchester and NIHR Manchester Biomedical Research Centre, Manchester, United Kingdom; ^6^Department of Medicine-DIMED, University of Padova, Padova, Italy; ^7^Department of Medicine, University of Kentucky, Kentucky, KY, United States

**Keywords:** systemic sclerosis, malnutrition, adipokines, outcome research, autoimmune disease

## Abstract

**Objective:** Malnutrition is a severe complication in Systemic Sclerosis (SSc) and it is associated with significant mortality. Notwithstanding, there is no defined screening or clinical pathway for patients, which is hampering effective management and limiting the opportunity for early intervention. Here we aim to identify a combined index predictive of malnutrition at 12 months using clinical data and specific serum adipokines.

**Methods:** This was an international, multicentre observational study involving 159 SSc patients in two independent discovery (*n* = 98) and validation (*n* = 61) cohorts. Besides routine clinical and serum data at baseline and 12 months, Malnutrition Universal Screening Tool (MUST) score and serum concentration of leptin and adiponectin were measured for each participant at baseline. The endpoint of malnutrition was defined according to European Society of Clinical Nutrition and Metabolism (ESPEN) recommendation. Significant parameters from univariate analysis were tested in logistic regression analysis to identify the predictive index of malnutrition in the derivation cohort.

**Results:** The onset of malnutrition at 12 months correlated with adiponectin, leptin and their ratio (A/L), MUST, clinical subset, disease duration, Scl70 and Forced Vital Capaciy (FVC). Logistic regression analysis defined the formula: −2.13 + (A/L^*^0.45) + (Scl70^*^0.28) as the best PREdictor of MAlnutrition in SSc (PREMASS) (AUC = 0.96; 95% CI 0.93, 0.99). PREMASS < −1.46 had a positive predictive value (PPV) > 62% and negative predictive value (NPV) > 97% for malnutrition at 12 months.

**Conclusion:** PREMASS is a feasible index which has shown very good performance in two independent cohorts for predicting malnutrition at 12 months in SSc. The implementation of PREMASS could aid both in clinical management and clinical trial stratification/enrichment to target malnutrition in SSc.

## Introduction

Systemic sclerosis (SSc) is a rare autoimmune disease characterized by microvascular injury, immune dysregulation and fibroblasts activation, leading to progressive skin and multi-organ fibrosis with potentially life-threatening complications (1). Systemic sclerosis is highly heterogeneous in clinical course for both type and severity of organ involvement. However, 75–98% of patients have at least one GI symptom (2, 3). When severe GI involvement occurs [up to 8% (4)], it confers a burden of 85% mortality within 9 years (5), accounting for 3–4% of overall mortality in SSc (6, 7). The prevalence of malnutrition in SSc is reported to be between 8 and 55% and it represents an independent risk factor for mortality (8–13). Many factors may contribute to the development of nutritional impairment in SSc, such as depression and anxiety, the severity of lung involvement, fatigue and myalgia, early satiety and microstomia, as well as the occurrence of digital ulcers or GI disturbances (13–18). Overall, there are no data on the efficacy of intervention in preventing or managing malnutrition since it has never been considered as main clinical outcome in randomized clinical trials for systemic sclerosis. This is in part due to the inability to define which patients are at risk of developing malnutrition in a time window targetable with a clinical trial, e.g., 12 months. Therefore, stratification for risk of malnutrition in SSc could aid both in clinical management and in the design of RCTs to determine efficacy in modifying this outcome.

Recently, the European Society of Clinical Nutrition and Metabolism (ESPEN), aiming at helping clinicians to document clinically relevant malnutrition by employing a clear definition, appointed a consensus group to provide criteria for the diagnosis of malnutrition (19).

The ESPEN consensus group delivered the following complementary definitions: a BMI <20 kg/m^2^ for subjects <70 years of age, and a BMI <22 kg/m^2^ for subjects ≥70 years older, combined with either a >5% weight loss over the last 3 months to cover for acute illnesses, or a >10% weight loss of habitual weight, independent of time, in order to include and be relevant for chronic conditions (19).

Weight loss is the main outcome in the longitudinal evaluation of malnutrition and it is inevitably accompanied by loss of adipose tissue. Besides energy storage, adipose tissue contributes to glucose and lipid metabolism control, through secretion of hormones, known as adipokines. Leptin and adiponectin are two major adipokines, playing a central role in metabolic homeostasis. Several studies have shown that leptin and adiponectin have opposing effects, supporting their use as a ratio, as reported in diabetes (20), coronary artery disease (21), and anorexia nervosa (22). A 25-years prospective study confirms that adiponectin to leptin ratio (A/L) is a predictor of metabolic profile change also in previously healthy individuals (23). Serum levels of adiponectin decrease with obesity and are positively associated with insulin sensitivity (24). On the other hand, leptin levels increase with body weight and suppress appetite by acting at the central nervous system level (25). In fact, leptin or leptin receptor deficiency induces a morbid obesity in both animals and humans (26, 27).

In SSc, two independent studies have shown that leptin levels correlate with BMI (28, 29). The trend of adiponectin, however, varies according to disease stage and remains controversial. Adiponectin is lower in the early diffuse form compared to late forms and inversely correlates with vital capacity, disease duration and skin involvement as measured by modified Rodnan skin score (30–34). A recent meta-analysis, despite the significant heterogeneity observed among the eligible studies, showed that adiponectin levels are significantly lower in SSc patients than normal controls, while on the contrary no significant differences in serum leptin levels were observed (28, 32, 35).

Once malnutrition occurs, it is difficult to reverse and worsens morbidity and mortality outcomes (7–9). To be able to inform studies for prevention of malnutrition based on stratified prognosis, here we aimed at identifying a combined clinical and biomarker-based index to stratify SSc patients for the risk of malnutrition according to the ESPEN definition in the next 12 months.

## Patients and Methods

### Design

International, multicentre, longitudinal, observational, parallel cohort study.

### Study Subjects

The discovery cohort comprised 110 consecutive patients with SSc, all fulfilling the EULAR/ACR 2013 criteria (36), and referred to the outpatient rheumatology services of the University Hospital of Messina (*n* = 60) and Padova (*n* = 50). An additional validation cohort of 70 SSc consecutive patients was enrolled within the observational study: Stratification for Risk of Progression in SSc (STRIKE–SSc) at the Scleroderma Research Program of the University of Leeds. Subjects engaged in weight loss program (*n* = 2) and malnourished according to the ESPEN criteria (*n* = 19) were excluded. Thus, the derivation cohort comprised 159 SSc patients (discovery cohort = 98; validation cohort = 61).

Each patient underwent clinical history and physical examination, and provided sociodemographic information. All patients were evaluated at study entry and at 12 months follow up visit.

This observational study was conducted following approval by the ethical committee of the University of Messina (prot. 15–15), University of Padova (prot. 2015–32) and the University of Leeds (NHS REC Approval number: STRIKE REC 15/NE/0211).

### Body Mass Index (BMI), Weight Loss and Malnutrition Assessment

In all participants, height and weight were measured by study personnel and used to calculate BMI (kg/m^2^), at baseline and at 12 months. Subjects were divided in the following groups: normal weight (BMI > 18.6 <24.9) and overweight/obese (BMI ≥ 25). Weight loss was calculated at 12 months in each patient and a ≥10% weight loss of baseline weight was used as outcome for the definition of malnutrition in combination with a BMI <20 kg/m^2^ or <22 kg/m^2^ if older than 70 years at 12 months, as defined by the ESPEN consensus (19).

### Malnutrition Universal Screening Tool

The malnutrition universal screening tool (MUST) provides a score based on the following parameters: body mass index (BMI): >20.0 = 0, 18.5–20 = 1, <18.5 = 2; weight loss score (unplanned weight loss in the past 3–6 months): <5% = 0; 5%−10% = 1: >10% = 2. The MUST also adds a score of 2 if there has been or is likely to be no nutritional intake for the next 5 days or more.

These scores are summed for the total score, conferring the degree of the risk for malnutrition: a score of 0 corresponds to low risk, 1 is moderate, ≥2 is high (37).

### Assessment of Circulating Adipokines Levels

Adiponectin and leptin serum levels were measured using anonymized bar-coded serum samples at baseline and at 12 months. All the tested compounds were quantitatively measured using an enzyme-linked immunosorbent assay kit (Abcam, Cambridge, UK) in the discovery cohort and with a bead-based multiplexed immunoassay (Human DiscoveryMAP® v.3.3, Myriad RBM, Austin, Texas, USA) in the validation cohort.

### Study End Points and Outcome Measures

Disease duration was calculated from the onset of the first non-RP symptom. Clinical subset of disease was defined according to Le Roy et al. (38). Forced vital capacity (FVC), total lung capacity (TLC), and lung diffusion capacity of carbon monoxide (DLCO) were analyzed, as previously described (39). Interstitial lung disease (ILD) was defined by DLCO and FVC <80% of the predicted values plus bibasilar fibrosis on High Resolution CT (HRCT). Pulmonary arterial hypertension (PAH) was confirmed by right heart catheterization (40), performed when indicated to confirm the diagnosis according to approved guidelines (41).

The primary end point was the development of malnutrition at 12 months using the recent definition of the ESPEN consensus as the combination of a BMI <20 kg/m^2^ for subjects <70 years of age, and a BMI <22 kg/m^2^ for subjects aged 70 years and older, and a weight loss ≥10% of baseline weight at 12 months or a BMI ≤ 18.5 (19).

### Statistical Analysis

The normal distribution of each variable was assessed using the Shapiro-Wilk test.

Summary results are expressed as the mean ± standard deviation for normally distributed variables, while the median with 95% confidence intervals was used for non-normally distributed variables, and relative frequencies for qualitative variables. The statistical analysis was performed using ANOVA for repeated measures for normally distributed variables or through the Friedman test for non-normally distributed variables. Comparison between percentages have been performed according to the “n-1” Chi-squared test for comparison between percentages. Univariate linear regression and multivariate logistic regression analysis were used accordingly.

To assess predictive ability receiver operating characteristic (ROC) curves were constructed using library (pROC). The area under the receiver operating characteristic curve (AUC) was estimated and a 95% CI determined using bootstrap resamples. AUCs were compared using a bootstrap significance test with the significance of differences between bootstrap AUCs assessed using a normal approximation. Data-derived optimum cut-points were selected by optimizing the sensitivity and the specificity. Where the predictive ability of a combination of variables was being assessed, multivariable logistic regression models were constructed and fitted probability values used in the ROC analysis as described. Analysis was undertaken using SPSS (version 24) for statistical computing.

## Results

### Clinical Features of Patients, Cohort Determination, and Prevalence of Organ Involvement

One hundred fifty-nine consecutive SSc patients, 98 from the discovery cohort, and 61 from the validation cohort, participated in the study. Clinical summary of the SSc patients is shown in Table 1. Discovery and validation cohorts were similar and comparable, apart from a lower frequency of anticentromere antibodies (ACA) in the discovery cohort. This is consistent with the autoantibody lookout of Italian patients, as previously described (42).

**Table 1:** Epidemiological and clinical features of the SSc study cohort.

	**Discovery cohort**	**Validation cohort**	***p***
Total group, no.	98	61	
Disease subset, D/L, no. (%)	34 (35)/64 (65)	23 (37)/45 (63)	0.79
Age, median (95% CI), years	56 (54–58)	55 (53–59)	0.85
Women, no. (%)	92 (93)	56 (91)	0.97
RP duration, median (95% CI), years	10 (8–16)	11 (8–15)	0.56
Disease duration (onset of first non-RP symptoms to baseline visit), median (95% CI)	7 (4–10)	7 (4–11)	0.64
mRSS for diffuse form, mean ± SD	15.6 ± 6.2	15.9 ± 7.2	0.78
mRSS for limited form, mean ± SD	4.3 ± 3.8	4.1 ± 3.6	0.74
Pulmonary fibrosis, no. (%)	34 (34)	19 (31)	0.69
TLC % predicted, mean ± SD	79 ± 21	78 ± 25	0.78
DLco % predicted, mean ± SD	67 ± 19	66 ± 16	0.73
FVC % predicted, mean ± SD	86 ± 21	84 ± 24	0.58
PAH, no. (%)	7 (7)	4 (6)	0.80
CK, mean ± SD	145 ± 48.4	135 ± 44.6	0.19
ANA+, no. (%)	98 (100)	61 (100)	1
ACA+, no. (%)	19 (19)	22 (36)	0.03
Scl70+, no. (%)	20 (20)	11 (18)	0.75
RNA III+, no. (%)	9 (9)	5 (8)	0.82
PM-Scl, no. (%)	5 (5)	3 (5)	1.00
BMI, mean (range)	23.4 (20.1–32.5)	23.1 (20.2–36.3)	0.71
Adiponectin, mean ± SD	6.2 (0.6–18.2)	5.5 (1.1–15.9)	0.28
Leptin, mean ± SD	21.6 (2.1–96.4)	19.3 (2.5–110)	0.41
MUST, median (range)	1 (0–3)	1 (0–4)	1
MUST = 0, no. (%)	44 (45)	29 (47)	0.8
MUST = 1, no. (%)	36 (37)	21 (34)	0.7
MUST ≥ 2, no. (%)	18 (18)	11 (18)	1
Immunosuppressors, no. (%)	45 (46)	24 (39)	0.38
Corticosteroids, no. (%)	28 (28)	14 (23)	0.48
GERD, no. (%)	73 (74)	48 (78)	0.56
Hiatal hernia, no. (%)	20 (20)	12 (19)	0.87
Gastritis, no. (%)	22 (22)	14 (23)	0.88
Costipation, no. (%)	12 (12)	6 (10)	0.69
Diarrhea, no. (%)	8 (8)	4 (6)	0.63
Esophagitis, no. (%)	9 (9)	5 (8)	0.82
Barrett's esophagus, no. (%)	4 (4)	4 (6)	0.56
Proctitis, no. (%)	4 (4)	2 (3)	0.74
PPI, no. (%)	77 (78)	48 (78)	0.88
Prokinetics, no. (%)	40 (40)	20 (32)	0.31
Antacids, no. (%)	24 (24)	14 (23)	0.88

*ACA, anticentromere antibodies; ANA, antinuclear antibodies; D, diffuse cutaneous SSc; L, limited cutaneous SSc; Dlco, diffusion capacity for carbon monoxide; GERD, Gastroesophageal reflux disease; FVC, forced vital capacity; CK, creatine kinase; mRSS, modified Rodnan skin score; PAH: Pulmonary Arterial Hypertension; PM-Scl, anti-PM-Scl antibodies; PPI, proton pump inhibtors; RNA III, anti-RNA polymerase III antibodies; RP, Raynaud's Phenomenon; Scl70, anti-topoisomerase I antibodies; SSc, systemic sclerosis; TLC, total lung capacity; BMI, body mass index; MUST, malnutrition universal screening tool*.

No statistical differences were observed in BMI, MUST, adiponectin, and leptin serum levels between discovery cohort and validation cohort at baseline, nor when analyzed according to age or gender. The lack of significant differences in the adiponectin and leptin was particularly important given the two methods used for analysis. Supplementary Table 1 shows the changes over time of the outcomes of interest for both discovery and validation cohort. After 12 months, BMI decreased over time (*p* < 0.001) while MUST increased in both cohorts. Accordingly, serum levels of leptin significantly decreased (*p* < 0.001) while adiponectin and adiponectin to leptin ratio (A/L) increased significantly (Supplementary Figure 1).

### Association Between BMI and Adiponectin to Leptin Ratio

**Figure 1:**
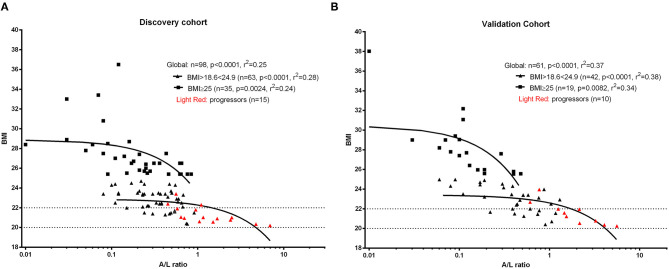
Linear regression analysis shows the association between body mass index (BMI) and adiponectin to leptin ratio (A/L ratio) at baseline for discovery cohort **(A)** and validation cohort **(B)**. A significant inverse correlation was observed between A/L and BMI in both cohorts. Red symbols show progressors (patients developing malnutrition at 12 months). The x axis (A/L ratio) was stretched to a logarithmic scale (log10) in both panels. Dotted lines represent the BMI cut-off according to age (BMI <20 kg/m^2^ if age <70 years or <22 kg/m^2^ if age >70 years) for the identification of future malnutrition at 12 months according to the European Society for Clinical Nutrition and Metabolism (ESPEN) definition.

Firstly, we observed that adiponectin and leptin serum levels were inversely correlated, as expected (28, 29) (*p* < 0.0001, r = −0.69, discovery cohort; *p* < 0.0001, r = −0.73, validation cohort). Next, we evaluated the distributi on of adiponectin and leptin according to BMI clustering in both discovery and validation cohorts and we found a similar distribution for both adiponectin and leptin across different BMI clusters (Supplementary Figure 1). Thus, we decided to use the A/L ratio to evaluate its association with BMI and we observed that A/L ratio significantly correlated with BMI clusters both in discovery and validation cohort (Figures 1A,B).

### MUST Performance in Predicting Malnutrition at 12 Months

**Figure 2:**
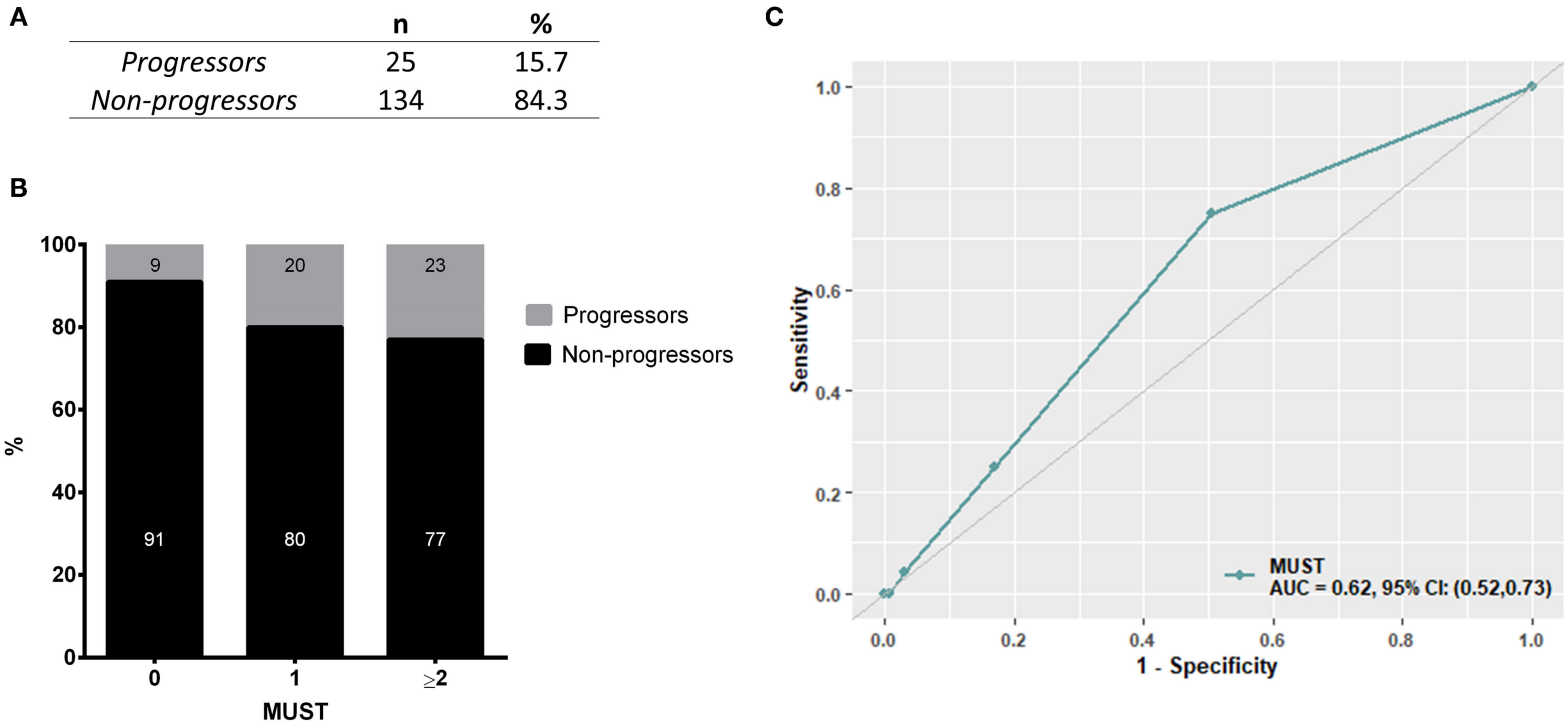
In the derivation cohort, resulting from the combination of the discovery cohort and the validation cohort, 15.7% of patients (*n* = 25, **A**) developed malnutrition at 12 months (progressors). Of note, 29% of patients having a low to moderate MUST score experienced malnutrition at 12 months and, on the other side, 77% of patients having a high risk of malnutrition (MUST ≥ 2) did not develop malnutrition at 12 months **(B)**. **(C)** shows the performance of MUST in predicting malnutrition at 12 months in the derivation cohort.

As initial analysis, we aimed to assess the profile of SSc patients developing malnutrition (progressors) at 12 months in both cohorts. The number of progressors was 15 (15.3%) in the discovery cohort and 10 (16.3%) in the validation cohort. Thus, 25 SSc patients among 159 (15.7%) met the end point of becoming malnourished at 12 months (Figure 2A). Furthermore, 9% of SSc patients having no malnutrition risk (MUST score = 0) and 20% of SSc patients having a moderate risk of malnutrition (MUST score = 1) at baseline still developed malnutrition at 12 months, while 77% of SSc patients with a high risk to develop malnutrition according to the MUST scoring system did become malnourished at 12 months (Figure 2B). The ability of MUST to predict malnutrition at 12 months was fair (AUC 0.62; 95% CI: 0.52960.73) (Figure 2C).

### Clinical Features of SSc Patients Developing Malnutrition

Next, we aimed to identify the discriminants associated with the risk of developing malnutrition in the discovery cohort by univariate analysis. Odds ratios with relative confidence intervals and statistical significance are shown in Supplementary Table 2. The factors associated with the development of malnutrition were shorter disease duration, a higher A/L ratio, a MUST score ≥ 2 and a higher mRSS and the diffuse form. Additionally progressors to malnutrition were more frequently Scl70 positive and had lower FVC and TLC at baseline. Next, we performed univariate analysis in the validation cohort and we observed similar results apart from TLC (Supplementary Table 3).

### Identification and Performance of the PREdictor of Malnutrition in Systemic Sclerosis (PREMASS) Index in Derivation Cohort

Next we combined both discovery and validation cohorts in a single cohort, hereafter defined derivation cohort. Univariate analysis for derivation cohort, reported in Table 2, confirmed the significance for adiponectin, leptin A/L, FVC, mRSS, Scl70, disease duration, diffuse subset of SSc and MUST score ≥2.

**Table 2:** Univariate logistic regression for baseline factors associated with the development of malnutrition at 12 months in derivation cohort.

**Predictor**	**Levels**	**OddsRatio**	**CI**	***p*-value**
Gender (Ref = male)	Female	1.01	(0.3, 4.6)	0.99
Age	(–)	1.00	(0.96, 1)	0.82
MUST (Ref = 0)	1	2.98	(1.1, 9.1)	0.4
	≥2	2.94	(0.43, 18)	0.02
MUST (numeric)	(–)	1.53	(0.94, 2.5)	0.12
Adiponectin	(–)	1.63	(1.4, 2)	<0.01
Leptin	(–)	0.80	(0.71, 0.87)	<0.01
A/L	(–)	18.38	(6.3, 69)	<0.01
Fibrosis (chest HRCT)	Y	2.38	(0.99, 5.9)	0.07
FVC%	(–)	0.94	(0.91, 0.96)	<0.01
TLC%	(–)	0.98	(0.96, 1)	0.13
DLCO%	(–)	0.99	(0.97, 1)	0.5
mRSS	(–)	1.13	(1.1, 1.2)	<0.01
Scl70	+ve	12.10	(4.6, 33)	<0.01
Disease duration (from non-RP)	(–)	0.84	(0.74, 0.92)	<0.01
Clinical subset (Ref = Diffuse)	Limited	0.32	(0.13, 0.78)	<0.01
PAH	Yes	0.81	(0.12, 3.2)	0.79
CK	(–)	1.00	(0.99, 1)	0.84

*A/L, adiponectin to leptin ratio; CK, creatine kinase; DLCO, Diffusion Lung CO; FVC, Forced Vital Capacity; HRCT, high resolution chest toography; MUST, Malnutrition Universal Screening Tool; mRSS, modified Rodnan skin score; nonRP, first non-Raynaud's disease manifestation; PAH, Pulmonary Arterial Hypertension; Scl70, antitopoisomerase I antibody; +ve, positive; TLC, Total Lung Capacity*.

**Figure 3:**
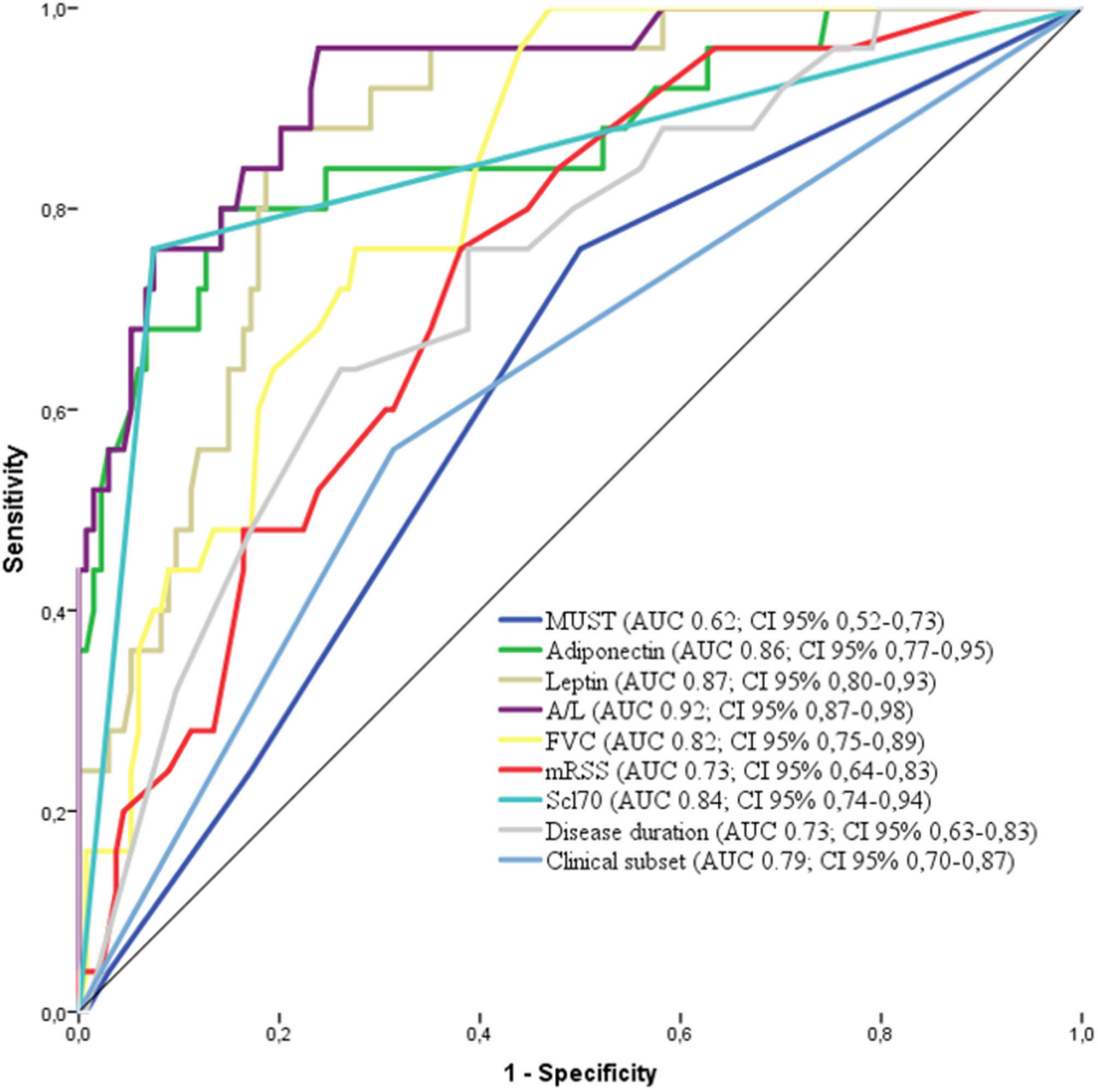
Receiver operating characteristics (ROC) curve for the variables associated with malnutrition in the derivation cohort [adiponectin, leptin, MUST, A/L, FVC, Scl70, clinical subset, and disease duration. AUC and 95% CI are reported for each variable in the legend. MUST, Malnutrition Universal Screening Tool; A/L, adiponectin to leptin ratio; FVC, Forcev Vital Capacity; Scl70, antitopoisomerase I antibodies.

The factors deemed significant in univariate analysis were analyzed by receiver operating curves (ROC) adopting progression to malnutrition as state variable. The best performing factors are shown in Figure 3. Firstly, we observed that the performance of A/L was superior to adiponectin or leptin alone (AUC: 0.92 vs. 0.86 for adiponectin–0.87 for leptin) and also superior to MUST (AUC: 0.92 vs. 0.62). Among the disease-specific variables, the presence of Scl70 showed the higher AUC (0.84), followed by lower FVC (0.82), diffuse form of clinical subset (0.79), shorter disease duration (0.73), and higher mRSS (0.73).

These 9 factors were further fitted in a logistic regression model adopting progression to malnutrition as binary outcome (malnutrition at 12 months =1 vs. no malnutrition = 0). The model was restricted to A/L and Scl70 as the 2 most significant variables according to the number of events (25 progressors).

**Figure 4:**
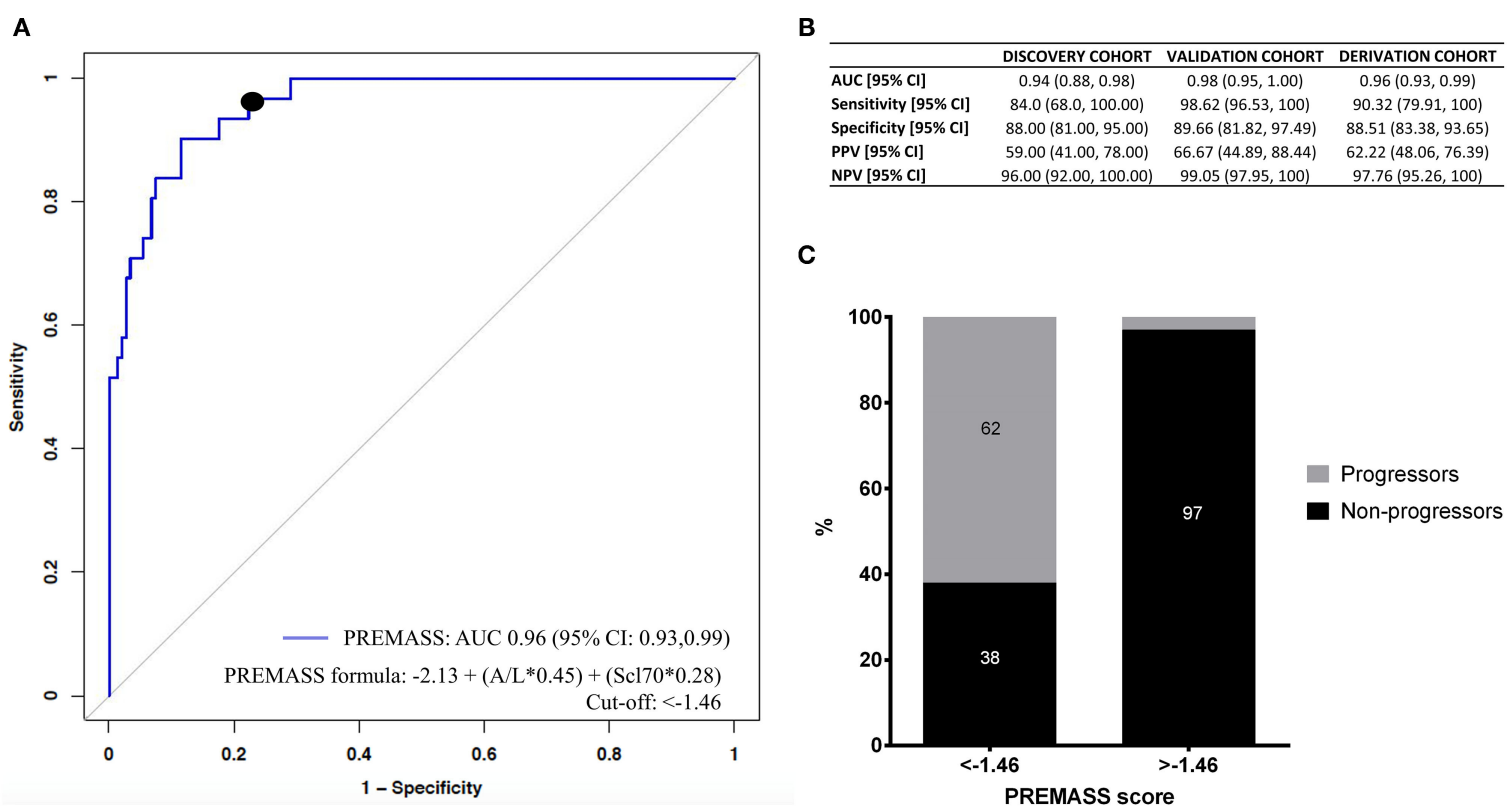
Receiver operating characteristics (ROC) curve **(A)** demonstrates that the best performance in predicting malnutrition at 12 months in the derivation cohort was represented by a combined index based on adiponectin to leptin ratio (A/L) and antitopoisomerase I antibodies (Scl70). Using the formula malnutrition = −2.13 + (A/L*0.45) + (Scl70*0.28), we validated the PREdictor of MAlnutrition in Systemic Sclerosis (PREMASS) index. **(B)** Shows the AUC, sensitivity, specificity, negative predictive value (NPV) and positive predictive value (PPV) for the discovery cohort, validation cohort, and derivation cohort. The percentage of progressors and non-progressors in the derivation cohort **(C)** are shown according to the cut-off (−1.46) of PREMASS optimized for sensitivity and specificity.

Accordingly, we built a combined index based on A/L and Scl70 (Figure 4A). Therefore, we identified as best predictor of malnutrition in systemic sclerosis (PREMASS) the formula: malnutrition at 12 months = −2.13 + (A/L^*^0.45) + (Scl70^*^0.28). A PREMASS score < -1.46, identified at maximizing for specificity and sensitivity, showed in the derivation cohort an AUC of 96% with 90% sensitivity [95% CI: 79–100] and 88% specificity (95% CI:83–93) for malnutrition at 12 months with an overall 62% positive predictive value (95% CI: 48–76) and 97% negative predictive value (95% CI: 95–100) (Figure 4B).

After dividing the derivation cohort according to the PREMASS cut-off (−1.46), we noted that, for patients with a score < -1.46, the PREMASS formula had a 62% PPV for becoming malnourished at 12 months, while for those having a score >-1.46 the formula had a 97% NPV (Figure 4C).

## Discussion

Malnutrition is an important severe clinical outcome in SSc, associated with significant mortality and to date remains poorly predictable with the available tools. The clear definition of malnutrition for complex populations, such as SSc, and the appropriate application of general tools, such as MUST, for its prediction remains unsatisfactory. Hence the identification of a tool to stratify patients with SSc for the risk of developing malnutrition would be particularly useful. Our longitudinal data suggest that in a population with 7 years average disease duration, 15.7% of SSc patients experience malnutrition at 12 months, according to the most updated classification of the ESPEN consensus in the absence of a SSc specific definition of malnutrition. A few reports have explored the value of the Malnutrition Universal Screening Tool (MUST) (11–13), and the Subjective Global Assessment (SGA) in assessing the level of malnutrition in SSc (43). However, no robust longitudinal studies have been performed to identify a reliable tool predictive of future malnutrition, which therefore remains an unmet need in SSc management (44). Low BMI is known to be an independent risk factor of malnutrition and for this reason is a major factor in the MUST formula. Notwithstanding, in our study, the performance of MUST in predicting malnutrition at 12 months was not satisfactory with 29% of SSc patients experiencing malnutrition at 12 months while having a none to low risk of malnutrition as assessed by the MUST score at baseline. MUST index has been developed for patients that develop malnutrition because of poor GI function (37). Our observation suggests that in SSc there are other factors, beyond GI function that can contribute to the development of malnutrition.

In fact, as already reported in other studies (45), we decided to avoid to focus only on a single BMI value (e.g., BMI <18.5) in the assessment of nutritional status in patients with SSc, considering more appropriate to identify SSc patients with malnutrition by employing the new ESPEN definition which includes a higher BMI cut-off in combination with weight loss. This approach provides a dynamic view of malnutrition and it has been consistently employed in recent research studies involving SSc patients (45, 46).

Independently of the cause of malnutrition, it is known that weight loss is associated with perturbances in the serum concentration of adipokines, the most studied of which are adiponectin and leptin, both in SSc and other conditions such as anorexia or diabetes (22, 24).

Therefore, we set out to measure adiponectin and leptin in our two SSc cohorts and test their value in predicting future malnutrition. Despite the limitation of testing adipokines serum levels with slightly different techniques, their concentrations were comparable between our discovery and validation cohorts. Most importantly, the ratio between the two neutralized the putative differences in concentration given by the two methodologies.

We noted that both adiponectin and leptin inversely correlated to BMI in SSc patients, as already reported (47). Further, we noticed that within the same BMI range, patients who became malnourished at 12 months had relatively higher A/L ratio compared to those who did not, suggesting that the serum levels of adiponectin and leptin may play a role in predicting future malnutrition.

Indeed, we noted that the increase in A/L after 12 months is due to an opposite change in adiponectin and leptin levels after 12 months in our SSc cohort. Previous studies suggest that adiponectin and leptin levels change in response to inflammatory cytokines, that can alter their levels over time (35). This has been reported in other studies whereas the production of adiponectin is down-regulated in response to pro-inflammatory cytokines and oxidative stress (48). In SSc, significant lower levels of adiponectin have been reported in very early active disease dcSSc (<18 months) compared to late disease subset (> 36 months) (31). It might be hypothesized that adiponectin and leptin levels vary according to disease activity in SSc, and their main regulator is skin disease progression, which tend to increase in the first 3 years of disease with self-limiting evolution.

Next, we aimed at identifying all the factors associated with malnutrition in order to build a composite score for the prediction of malnutrition. Our analysis showed that A/L and MUST were significantly associated with malnutrition. Additionally, malnourished SSc patients had the diffuse form of the disease, with higher mRSS and lower FVC, a shorter disease duration and were more frequently Scl70 positive. Among these, Scl70 positivity had the most relevant significance and it was included in our analysis, in association with A/L, for the identification of the most performing index. Scl70 positivity has been already associated with capillary rarefaction and digital ulcers development, severe skin involvement and ILD and also poorer prognosis in SSc patients, and our data further extends the relevance of these antibodies in the management and prediction of SSc-comorbidities (49).

Other circulating biomarkers have been studied in systemic sclerosis, such as prealbumin or hemoglobin, but there are no clear evidence that any of these factor are predictors of future malnutrition, and they could be considered markers of active malnutrition instead (11, 12, 50).

Our study has some limitations: the index has been validated to predict malnutrition at 12 months and longer longitudinal studies are needed to assess its validity for long-term changes. Furthermore, no additional circulating biomarkers of malnutrition were tested nor healthy participants were enrolled, thus not allowing us to assess the differences in adipokines serum levels in comparison to the general population. In addition, our cohorts correspond to consecutive patients under treatment with vasodilators and/or immunosuppressants (mycophenolate, methotrexate, or azathioprine), so the influence of different therapeutic regimens on adipokines levels might be difficult to ascertain. Lastly the absolute concentration of adiponectin and leptin could vary slightly among techniques, nevertheless the ratio between adiponectin and leptin could overcome this latter issue.

Using BMI as a metric for malnutrition has many well-known limitations and indeed, waist circumference measurement would be a better alternative. Nonetheless, the use of skin-dependent methods are not applicable in SSc research due to the intrinsic features of the disease.

In conclusion, malnutrition is frequent in SSc patients and specific clinical features define a subset of patients more susceptible to it. The PREMASS index, based on A/L and Scl70, is able to predict malnutrition in SSc, and it is objective and relatively easy to measure. Such an index could be useful in stratifying patients at risk of malnutrition for more intense intervention and/or nutritional support, and in clinical research to enrich for patients at risk of clinically relevant malnutrition in clinical trials that target malnutrition as objective. While the purpose of our study was to define the risk of malnutrition in the following 12 months, studies aimed at predicting the future onset of malnutrition in longer time frame may be useful for patient stratification. Future studies should also address the patient reported outcomes that are most affected by the onset of malnutrition. As putative disease-modifying drugs enter trials with necessarily smaller *n* (51), PREMASS indexing may contribute to risk stratification to balance randomization. Subsequent work in this area could perform interval malnutrition assessments to understand the factors that predict more rapid onset of malnutrition.

## Data Availability Statement

The raw data supporting the conclusions of this article will be made available by the authors, without undue reservation.

## Ethics Statement

The studies involving human participants were reviewed and approved by the respective ethical committees: University of Messina: prot. 15-15), University of Padova: prot. 2015-32 and University of Leeds: NHS REC Approval number (STRIKE REC 15/NE/0211). The patients/participants provided their written informed consent to participate in this study.

## Author Contributions

GB, FD, and WR: concept and design of the study. EP, AB, GP, DS, NI, EZ, GA, AF, and MW: data collection, data analysis, and interpretation. FS, MB, PE, SG, AS, and FC: manuscript preparation. All authors revised and approved the manuscript to be published.

## Conflict of Interest

The authors declare that the research was conducted in the absence of any commercial or financial relationships that could be construed as a potential conflict of interest.
