# Supplementary material for: The PREdictor of MAlnutrition in Systemic Sclerosis (PREMASS) Score: A Combined Index to Predict 12 Months Onset of Malnutrition in Systemic Sclerosis
Source: Front Med (Lausanne). 2021 Mar 17;8:651748. doi: 10.3389/fmed.2021.651748 (PMC8010181; doi:10.3389/fmed.2021.651748)
Supplement: Supplementary file 1 [file Data_Sheet_1.docx]

**Supplemental Table S1.**

**Body Mass Index, Malnutrition Universal Screening Tool and adipokines changes over time**

|  |  | **DISCOVERY COHORT**  **(n=98)** | | *p* | **VALIDATION COHORT**  **(n=61)** | | *p* |
| --- | --- | --- | --- | --- | --- | --- | --- |
|  |  | Baseline | 12 months |  | Baseline | 12 months |  |
| *BMI, median (range)* | | 23.4  (20.3-32.5) | 21.3  (18.4-37.1) | <0.001 | 23.1  (20.5-36.3) | 21.9  (18.7-38.1) | <0.001 |
| *Adiponectin (ug/ml), median (range)* | | 6.2  (0.6 - 18.2) | 7.1  (0.2 – 21.3) | <0.001 | 5.5  (1.1 - 15.9) | 6.9  (1.7 – 19.6) | <0.001 |
| *Leptin (ng/ml), median (range)* | | 21.6  (2.1 - 96.4) | 13.9  (0.6 - 91.3) | <0.001 | 19.3  (2.5 - 110) | 12.9  (0.7 - 107) | <0.001 |
| *A/L, median (range)* | | 0.31  (0.01 - 6.1) | 0.56  (0.003 – 8.4) | <0.001 | 0.39  (0.01 - 5.9) | 0.53  (0.03 - 7.9) | <0.001 |
|  | |  |  |  |  |  |  |
| *MUST, median (range)* | | 1 (0 - 3) | 1 (0 - 4) | 0.04 | 1 (0 - 4) | 1 (0 - 4) | 0.03 |
| *MUST = 0, no. (%)* | | 44 (45) | 33 (34) | 0.21 | 29 (47) | 22 (36) | 0.21 |
| *MUST = 1, no. (%)* | | 36 (37) | 42 (43) | 0.50 | 21 (34) | 24 (39) | 0.56 |
| *MUST ≥ 2, no. (%)* | | 18 (18) | 23 (23) | 0.49 | 11 (18) | 15 (25) | 0.34 |

*The Table shows body mass index (BMI), malnutrition universal screening tool (MUST) and adipokines changes at 12 months for both discovery cohort and validation cohort. BMI significantly decreased at 12 months in both cohorts, and MUST scores significantly increased, with less systemic sclerosis patients scoring 0 (low risk of malnutrition) and accordingly more patients scoring a MUST score ≥2 (high risk of malnutrition) at 12 months. Leptin significantly decreases while adiponectin and accordingly A/L increase at 12 months in both cohorts.*

**Supplemental Table S2. Univariate logistic regression for baseline factors associated with the development of malnutrition at 12 months in the discovery cohort (n=98)**

| **Predictor** | **Levels** | **Odds ratio** | **95% CI** | **p-value** |
| --- | --- | --- | --- | --- |
| *Gender(ref=Male)* | F | 0.75 | (0.22, 3.39) | 0.67 |
| *AGE* | (-) | 0.93 | (0.97, 1.11) | 0.16 |
| *MUST (ref=0)* | 1 | 1.90 | (0.53, 7.12) | 0.28 |
|  | ≥2 | 3.64 | (0.93, 14.4) | 0.05 |
| *MUST (numeric)* | (-) | 1.81 | (1.02, 3.27) | 0.10 |
| *Adiponectin* | (-) | 1.78 | (1.18, 1.97) | <0.01 |
| *Leptin* | (-) | 1.35 | (0.70, 1.46) | <0.01 |
| *A/L* | (-) | 10.6 | (3.18, 38.5) | <0.01 |
| *Chest HRCT fibrosis* | Y | 2.54 | (0.99, 7.44) | 0.06 |
| *FVC%* | (-) | 0.96 | (0.91, 1.24) | <0.01 |
| *TLC%* | (-) | 0.93 | (0.92, 1.14) | 0.03 |
| *DLCo%* | (-) | 0.99 | (0.97, 1.01) | 0.34 |
| *mRSS* | (-) | 1.07 | (0.99, 1.15) | 0.05 |
| *Scl70* | +ve | 14.7 | (4.23, 48.4) | <0.01 |
| *Disease duration (from nonRP)* | (-) | 0.88 | (0.71, 0.97) | 0.01 |
| *Disease subset (ref=diffuse)* | Limited | 0.43 | (0.16, 1.10) | 0.02 |
| *PAH* | Y | 1.71 | (0.43, 5.64) | 0.39 |
| *CK* | (-) | 1.00 | (1.00, 1.01) | 0.49 |

A/L: adiponectin to leptin ratio; CK: creatine kinase; DLCo: Diffusion Lung carbon monoxide; FVC: Forced Vital Capacity; HRCT: High resolution Computed Tomography; MUST: Malnutrition Universal Screening Tool; mRSS: modified Rodnan skin score; nonRP: first non-Raynaud's disease manifestation; PAH: Pulmonary Arterial Hypertension; Scl70: antitopoisomerase I antibody; TLC: Total Lung Capacity; +ve= positive

**Supplemental Table S3. Univariate logistic regression for baseline factors associated with the development of malnutrition at 12 months in the validation cohort (n=61)**

| **Predictor** | **Levels** | **Odds ratio** | **95% CI** | **p-value** |
| --- | --- | --- | --- | --- |
| *Gender(ref=Male)* | F | 0.85 | (0.33, 3.12) | 0.70 |
| *AGE* | (-) | 0.95 | (0.91, 1.14) | 0.19 |
| *MUST (ref=0)* | 1 | 2.12 | (0.44, 5.97) | 0.15 |
|  | ≥2 | 4.21 | (0.67, 16.2) | 0.05 |
| *MUST (numeric)* | (-) | 1.91 | (1.01, 3.56) | 0.23 |
| *Adiponectin* | (-) | 2.32 | (1.67, 3.42) | <0.01 |
| *Leptin* | (-) | 1.97 | (0.89, 3.16) | <0.01 |
| *A/L* | (-) | 12.8 | (4.18, 41.7) | <0.01 |
| *Chest HRCT fibrosis* | Y | 2.34 | (0.87, 6.27) | 0.07 |
| *FVC%* | (-) | 1.36 | (1.01, 2.67) | <0.01 |
| *TLC%* | (-) | 0.97 | (0.88, 1.54) | 0.08 |
| *DLCo%* | (-) | 1.18 | (0.87, 1.45) | 0.45 |
| *mRSS* | (-) | 2.47 | (1.45, 3.85) | 0.01 |
| *Scl70* | +ve | 23.5 | (8.23, 54.4) | <0.01 |
| *Disease duration (from nonRP)* | (-) | 0.94 | (0.86, 0.99) | <0.01 |
| *Disease subset (ref=diffuse)* | Limited | 0.73 | (0.46, 1.34) | <0.01 |
| *PAH* | Y | 1.81 | (0.76, 3.64) | 0.64 |
| *CK* | (-) | 1.00 | (1.00, 1.02) | 0.65 |

A/L: adiponectin to leptin ratio; CK: creatine kinase; DLCo: Diffusion Lung carbon monoxide; FVC: Forced Vital Capacity; HRCT: High resolution Computed Tomography; MUST: Malnutrition Universal Screening Tool; mRSS: modified Rodnan skin score; nonRP: first non-Raynaud's disease manifestation; PAH: Pulmonary Arterial Hypertension; Scl70: antitopoisomerase I antibody; TLC: Total Lung Capacity; +ve= positive

**Supplemental Figure S1.**

*
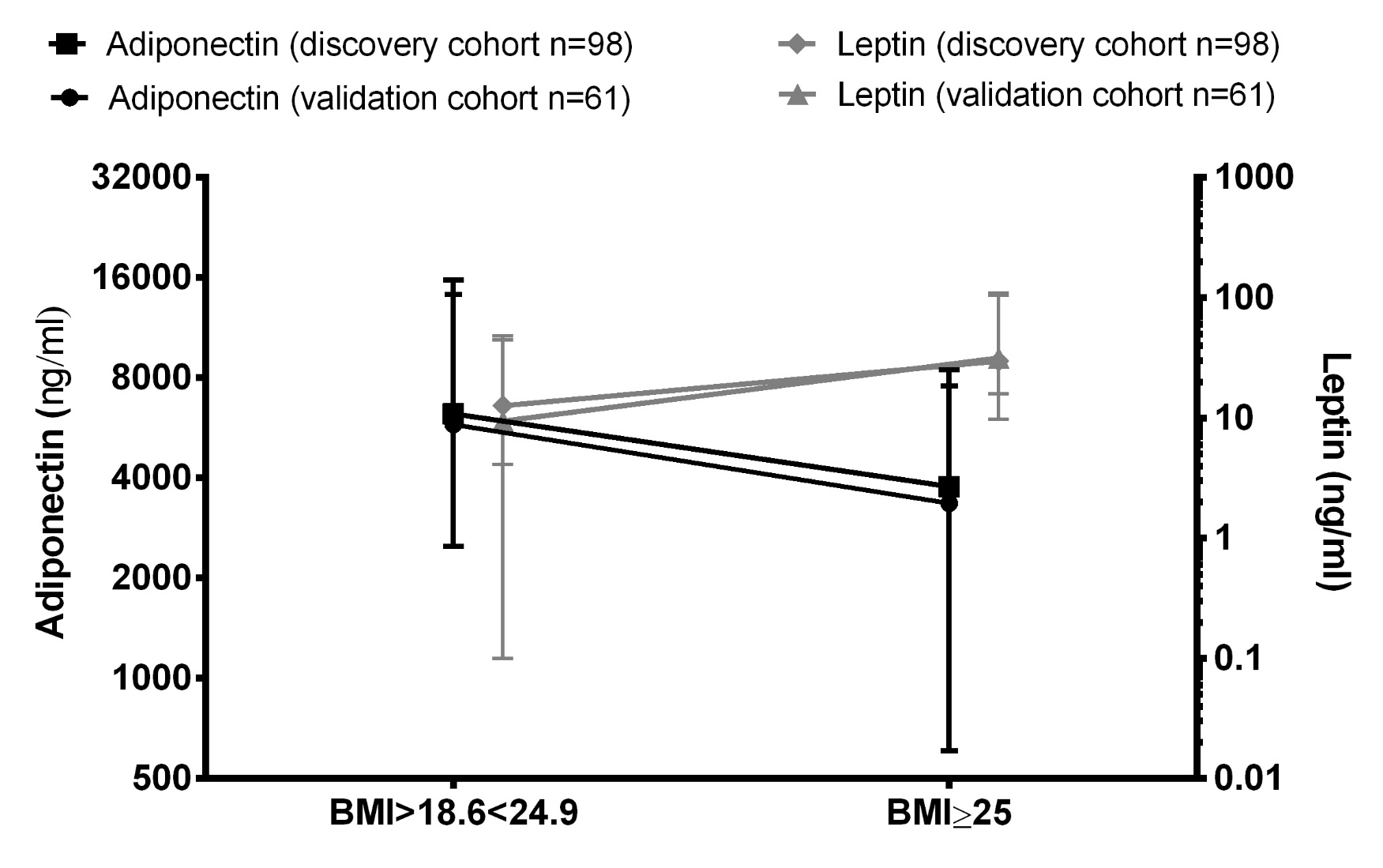
*

*The figure shows that at baseline adiponectin (plotted on left ordinate as log 2) and leptin (plotted on the right ordinate as log10) associate differently (data shown as median ± SE) when grouped by BMI clusters in normal weight (BMI >18.6<24.9 kg/m^2^) and overweight/obese (BMI ≥ 25 kg/m^2^) both in the discovery and in the validation cohort.*
